# Persisting predominant clinical CC87 *Listeria monocytogenes* in China: evolutionary dynamics and diversification

**DOI:** 10.1080/22221751.2026.2688488

**Published:** 2026-06-12

**Authors:** Chaohui Huang, Xiaoying Qin, Guochen Xu, Ruyi Xia, Han Han, Zegang Wang, Xiangyu Deng, Xin'an Jiao, Shaoting Li, Yuelan Yin

**Affiliations:** a College of Bioscience and Biotechnology, Yangzhou University, Yangzhou, People’s Republic of China; b Key Laboratory of Prevention and Control of Biological Hazard Factors (Animal Origin) for Agrifood Safety and Quality, Ministry of Agriculture and Rural Affairs of the People's Republic of China, Yangzhou University, Yangzhou, People’s Republic of China; c Jiangsu Co-innovation Center for Prevention and Control of Important Animal Infectious Disease and Zoonoses, Yangzhou, People’s Republic of China; d School of Biomedical and Pharmaceutical Sciences, Guangdong University of Technology, Guangzhou, People’s Republic of China; e Center for Food Safety, University of Georgia, Griffin, GA, USA

**Keywords:** Listeriosis, *Listeria monocytogenes*, hypervirulent CC87, evolutionary dynamics, WGS based surveillance system

## Abstract

Listeriosis, caused by *Listeria monocytogenes* (*L*. *monocytogenes*), is a severe foodborne infection linked to neonatal meningitis and high mortality. However, long-term epidemiological trends in China remain undercharacterized. We conducted a 60-year literature-based review of listeriosis cases in China (1961–2020) and integrated publicly available genomic data from Chinese clinical *L*. *monocytogenes* isolates to explore molecular epidemiology and evolutionary patterns. Our analysis identified three distinct epidemiological periods: Sentinel (–1999), Growth (2000–2009), and High-Incidence Plateau (2010–2020). Overall, the case fatality rate was high (24.3%), particularly in perinatal cases (33%), with significant pregnancy complications, including miscarriage (35%) and neonatal mortality (27.5%). Spatial clustering was observed in economically developed regions, likely reflecting improved surveillance and increased consumption of ready-to-eat foods. Genomic analysis revealed that the CC87 clone, carrying both LIPI-1 and LIPI-4, forms a distinct evolutionary branch in China. Bayesian phylodynamic analysis estimated that the global CC87 lineage originated around 1967 (median MRCA), with a gradual increase in effective population size from the late 1980s, indicating prolonged national expansion and recent stabilization. Phylogenetic and genomic analyses revealed weak phylogeographic structure within China, with extensive inter-provincial mixing and minimal local clustering. These findings suggest widespread nationwide circulation, frequent cross-regional transmission, and ongoing diversification of CC87. By integrating epidemiological and genomic data, our study provides a comprehensive overview of listeriosis dynamics in China, highlighting hypervirulent clones as critical targets for public health interventions and informing One Health–oriented prevention strategies.

## Introduction

Listeria *monocytogenes* is a formidable foodborne zoonotic pathogen with a ubiquitous environmental distribution and a remarkably broad host range [1]. Pregnant women, neonates, and immunocompromised individuals are at particularly high risk. Infection can progress to severe invasive listeriosis, manifesting as septicaemia, meningitis, meningoencephalitis, or pregnancy-associated complications such as spontaneous abortion and neonatal sepsis. The disease is associated with a high case-fatality rate (20–30%) and the highest hospitalization rate among all foodborne illnesses [2]. Large-scale outbreaks have repeatedly highlighted the profound public health impact of *L*. *monocytogenes*, including the 2017 South African outbreak linked to ready-to-eat processed meats (1060 confirmed cases and 216 deaths) [3], and the 1992 French outbreak associated with pork tongue in aspic (279 infections and 85 deaths) [4]. Despite routine active surveillance in North America and Europe, recurrent outbreaks continue, predominantly involving contaminated animal-derived foods [5–7]. This persistence highlights the substantial and enduring public health and economic burden of *L*. *monocytogenes*. Moreover, the epidemiology of listeriosis remains highly dynamic, exhibiting marked spatiotemporal heterogeneity shaped by regional ecological factors, socioeconomic determinants, and divergent public health policies.

The profound socioeconomic transformation in China over recent decades has revealed a range of emerging public health concerns, which not only significantly alter its food production and consumption patterns but have also introduced a range of new public health challenges [8–10]. However, threats such as listeriosis remained largely peripheral to mainstream scientific and public health priorities, and research on the disease was limited to sporadic publications spanning the three decades leading up to 2000 [11]. Since the early 2000s, rapid urbanization and economic growth have fundamentally reshaped lifestyles and dietary patterns. These changes are evident in the expansion of centralized food processing systems, the frequent skipping of final heating steps, and increasing rates of food consumption outside the home [12–14]. These combined factors have significantly increased the risk of listeriosis transmission through the complex food supply chain. Despite these evolving risks, systematic scientific reporting on listeriosis remained fragmented well into the early twenty-first century. Consequently, reported cases of listeriosis in national surveillance systems have steadily increased since 2001. This shift has provided a solid epidemiological foundation for more systematic research and directly led to several key studies, such as the Chinese CDC's national molecular epidemiological analysis of 144 cases from 2008 to 2019 using MLST typing [15], and a comprehensive evaluation by China National Center for Food Safety Risk Assessment (CFSA) of dietary risk factors in 359 cases between 2013 and 2022 [16]. The rapid socioeconomic changes in China have increased the risk of listeriosis transmission, leading to a rise in reported cases and prompting key epidemiological research.

Despite prior progress in understanding the epidemiology and molecular characteristics of *L*. *monocytogenes* in China, research remains largely regional or city-specific studies [17–20], lacking an integrated national framework. To date, only two broad literature-based syntheses have attempted to elucidate long-term national trends in clinical and epidemiological characteristics. Feng et al. (1964–2010; 147 cases) and Chen et al. (2008–2017; 759 cases) conducted decade-spanning reviews [21,22]. However, their findings diverged notably. Feng reported the highest disease burden among neonates (0–28 days) [21], whereas Chen identified children aged 2–10 years as the most affected group [22]. These inconsistencies likely reflect the differing timeframes, inclusion criteria, and data sources of the respective reviews, underscoring the inherent limitations and temporal biases in reconstructing a coherent national narrative from fragmented literature. Although substantial progress has been made in China in outbreak investigations and molecular subtyping of *L*. *monocytogenes*, a comprehensive, long-term national surveillance framework remains incomplete, limiting our understanding of the spatiotemporal dynamics and evolutionary trends of listeriosis. More importantly, molecular epidemiology has identified clonal complex 87 (CC87) as the predominant lineage responsible for invasive infections in China and across East Asia [11,22,23]. Nevertheless, the population dynamics of this clone throughout its long-term circulation in China, as well as the evolutionary forces driving its persistence and adaptation, remain largely unresolved.

This study represents the first comprehensive integration of bibliometric and systematic analyses to map the epidemiological landscape of listeriosis in China from 2000 to 2020, a period marked by rapid socioeconomic transformation and strengthening public health infrastructure. By placing epidemiological trends within this broader societal context, we highlight the social and systemic factors, including enhanced surveillance and diagnostic capacity, that have shaped temporal shifts in case reporting. Building on this perspective, we examine long-term spatiotemporal and demographic patterns of listeriosis, assess how surveillance capacity, evolving food industry practices, and dietary behaviours have influenced epidemic trends, and elucidate the transmission dynamics and clinical impact of the dominant clone CC87. Together, these analyses provide a rigorous historical and scientific foundation for developing tailored, evidence-based strategies to prevent and control listeriosis in China.

## Materials and methods

### Source of materials

To investigate the epidemic patterns of *L*. *monocytogenes* infections in China, a comprehensive literature search was conducted across multiple databases, including China National Knowledge Infrastructure (CNKI), Wanfang Academic Journal Database, VIP Journal Integration Platform, and the US National Center for Biotechnology Information (NCBI), Semantic Scholar, Web of Science, Cochrane Library, and Embase. Search terms included combinations of “*L*. *monocytogenes*,” “listeriosis,” “clinical listeriosis cases,” “Listerial infection,” “*Listeria* report,” “*Listeria* epidemiology,” “symptoms of Listeria infection,” and “predominant clonal complex types of *L*. *monocytogenes*.” Following the literature search, data on human listeriosis cases were extracted and analyzed to characterize epidemiological trends in the Chinese population. The search covered articles published between 1961 and 2020.

### Study inclusion and exclusion criteria

Studies that fulfilled the following a priori eligibility criteria were included: (1) All Listeria cases were sourced from China. (2) The articles described the clinical characteristics of listeriosis or studies on clinically isolated strains. (3) The geographic region of the patients. (4) Clinical features in perinatal cases (neonates, pregnant women, and fetuses) versus non-perinatal cases. (5) Serotype, sequence type (ST), and clonal complex (CC) group of the isolated strains. (6) Dietary habits of the patients. Studies that fulfilled the following a priori eligibility criteria were excluded: Duplicate case reports were removed based on basic patient information, age, and treatment outcomes.

### Data extraction

The initial literature search yielded 6700 records, of which, 3132 duplicates were excluded after reviewing titles and abstracts. Thus, 432 articles published between 1961 and 2020 were included in this review, comprising a total of 1299 cases. Among the 769 cases with known gender, female patients slightly outnumbered male patients (386 vs. 383), while gender was not reported in 284 cases. The majority of patients presented with sepsis (48%) and meningitis (26%). Among non-perinatal cases, 186 patients (20%) had severe immune-compromising conditions, including hematological/oncological disorders (39 cases), systemic lupus erythematosus (13 cases), diabetes (9 cases), heart disease (6 cases), and AIDS (2 cases).

### Definition and classification of cases

Patients from clinical reports were categorized into the following two groups based on their symptoms: (1) Perinatal cases: referring to pregnant women and their fetuses or neonates within 28 days after birth. (2) Non-perinatal cases: all cases other than those classified as perinatal. Pregnant women and their fetuses were counted separately. Based on clinical characteristics, cases were further classified into the following three categories: (1) Central nervous system (CNS) infection: defined either by the isolation of Listeria from the patient’s cerebrospinal fluid (CSF) or by the presence of neurological symptoms. (2) Sepsis: cases in which Listeria was detected in blood samples, and the physician did not diagnose a CNS infection. (3) Focal infection/Gastroenteritis: cases where Listeria was isolated only from non-blood and non-CNS specimens.

### Global phylogenetic reconstruction of CC87 isolates

To construct a global phylogeny of the CC87 isolates, all *L. monocytogenes* genomes available in NCBI Pathogen Detection as of October 2025 were analyzed using BIGSdb-Lm platform for clonal complex assignment [24]. In total, 1271 CC87 isolates were identified (Supplementary Table 1). The assembled genomes of the CC87 isolates were subject to cgMLST analysis against the BIGSdb-Lm cgMLST scheme using chewBBACA suite v2.5.5 [25]. The resulting cgMLST allelic profiles were used to build a minimum spanning tree (MST) using stand-alone GrapeTree v1.5.1 with MSTree V2 algorithm [26]. These CC87 isolates were used for further SNP-based phylodynamic analysis. Antimicrobial resistance genes were detected using ABRicate (https://github.com/tseemann/abricate) by screening against the Comprehensive Antibiotic Resistance Database (CARD), applying minimum thresholds of 80% sequence identity and 80% gene coverage. The virulence genes of *L. monocytogenes* were identified using ABRicate with 80% coverage and 80% nucleotide identity cutoff.

### Bayesian phylogenetic reconstruction

For Bayesian phylogenetic reconstruction, 646 representative CC87 genomes (Supplementary Table 2) were selected using a cgMLST-based subsampling strategy to reduce redundancy. First, isolates were grouped by country, year of collection, and source. Within each group, genomes were selected one by one in random order, and a genome was kept only if it was more than 10 cgMLST allelic differences away from all genomes already selected. This ensured that highly similar isolates were not repeatedly included, while maintaining broad representation across time, geography, and sources for subsequent phylodynamic analyses. Whole genome alignment of the representative CC87 isolates was performed using SKA2 [17]. The alignment was generated with the Gubbins script generate_ska_alignment.py [18], using the complete genome of *L. monocytogenes* FSL J1-175 (accession number CP062129.1) as the reference. Gubbins v3.3.1 [18] was then used to identify and filter recombinant regions from the alignment. Temporal signal among the isolates was assessed using TempEst v1.5.368 by performing root-to-tip regression analysis of genetic divergence against sampling dates. Bayesian phylogenetic reconstruction was subsequently performed on the recombination-filtered alignment of polymorphic sites using the Bayesian Evolutionary Analysis by Sampling Trees (BEAST) v1.10.4. Multiple BEAST analyses were conducted using constant population size, exponential growth, and Bayesian Skyline demographic priors, each tested with either a strict clock or an uncorrelated log-normal relaxed clock. The best-fitting model was an uncorrelated log-normal relaxed clock combined with a Bayesian Skyline coalescent prior, which was therefore used to infer time-calibrated phylogeny. A Markov chain Monte Carlo (MCMC) chain of 200 million iterations, with sampling every 10,000 steps, was run in three independent replicates, and the first 20 million trees of each run were discarded as burn-in. Maximum clade credibility trees were generated by Tree Annotator v1.10.4 and visualized with FigTree v1.4.4. Estimates of the time of the most recent common ancestor (MRCA) and substitution rates were estimated by Tracer v1.7.1. The Bayesian skyline plot was reconstructed in Tracer v1.7.1. A core-genome SNP alignment was generated from the recombination-filtered polymorphic sites, and pairwise genomic distances were computed using snp-dists (https://github.com/tseemann/snp-dists).

Among the 646 representative CC87 genomes, 267 isolates from China were further selected for a China-specific Bayesian phylogenetic reconstruction using the same alignment pipeline, recombination filtering procedure, and BEAST model parameters as described above. This separate analysis enabled higher-resolution inference of the evolutionary history and population dynamics of CC87 within China.

## Results

### Geographical and temporal endemicity of clinical listeriosis

Among the 1299 cases included from 1961 to 2020, cases were reported from 18 provinces, 4 municipalities, and the Taiwan region of China. Beijing reported the highest number of cases (n = 234, 18.0%), followed by Sichuan Province (n = 172, 13.2%), Jiangsu Province (n = 133, 10.2%), Taiwan (n = 132, 10.2%), Guangdong Province (n = 131, 10.1%), and Shanghai (n = 82, 6.3%) (Figure 1A). With the exception of Sichuan Province, most Provinces with a high number of cases were coastal (Figure 1B). The western region reported significantly fewer cases than the eastern region, and other inland areas had relatively low case detection rates, suggesting a regional endemic pattern of listeriosis in China.

**Figure 1.**
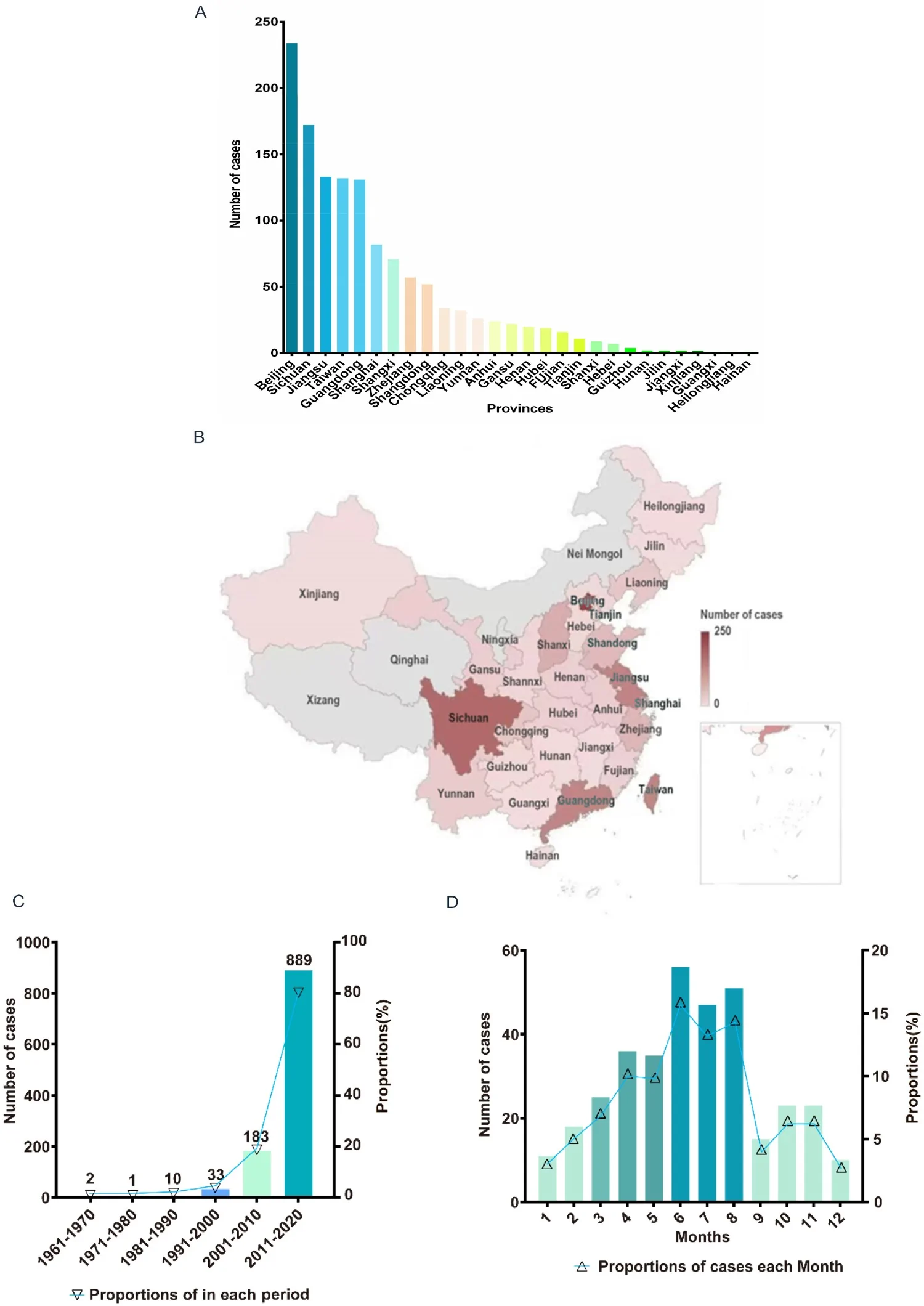
Geographical and Temporal distribution of clinical cases. (A) Reported Listeriosis cases by Province in China, 1961–2020; (B) geographic distribution of reported listeriosis in China; (C) human Listeriosis cases by Year; D: Human Listeriosis by month.

It is evident from Figure 1C that relatively few listeriosis cases were reported before 2000. From 1961 to 1970, only two cases were documented, one in a neonatal patient and one in an immunocompetent adult. Between 1971 and 1980, a single case was reported, followed by ten cases from 1981 to 1990, and 33 cases from 1991 to 2000. After 2000, the number of reported cases increased steadily, reaching 183 cases by 2010 and 889 cases between 2011 and 2020. These trends delineate three epidemiological phases of listeriosis in China: a sentinel period (1961–2000), a growth period (2001–2010), and a high-incidence plateau (2011–2020).

Among the 1299 reported cases, only 344 had clearly documented months of onset. Within this subset, listeriosis displayed a distinct and pronounced seasonal pattern, with case numbers from June to August significantly higher than in other months (Figure 1D). June represented the peak incidence, followed by spring months from March to May. This pattern is consistent with reports from the European Union, where listeriosis also peaks in summer. The cold-adapted *L*. *monocytogenes* poses a heightened summer risk of contaminating ready-to-eat foods, particularly refrigerated items such as Chinese cold dishes when they are not thoroughly reheated.

### Epidemiological trends of human listeriosis from 1961 to 2020

#### Case distribution by age and risk group

Among the 1299 reported listeriosis cases, 567 had clearly documented years of onset. Case distribution varied substantially across age groups and risk categories. Neonates, pregnant women and immunocompromised individuals aged ≥46 years together accounted for a major proportion of cases (9.17, 24.7 and 36.68%, respectively) (Figure 2A). Within the latter group, children aged 2–15 years had the highest number of cases. The 26–35-year and ≥56-year groups contributed the largest proportions overall (16.58% and 19.05%, respectively) (Figure 2A), with the former driven by pregnancy-associated infections. Taken together, these patterns highlight the recognised vulnerability of neonates and immunocompromised individuals, reflecting both physiological susceptibility and differential exposure risks.

**Figure 2.**
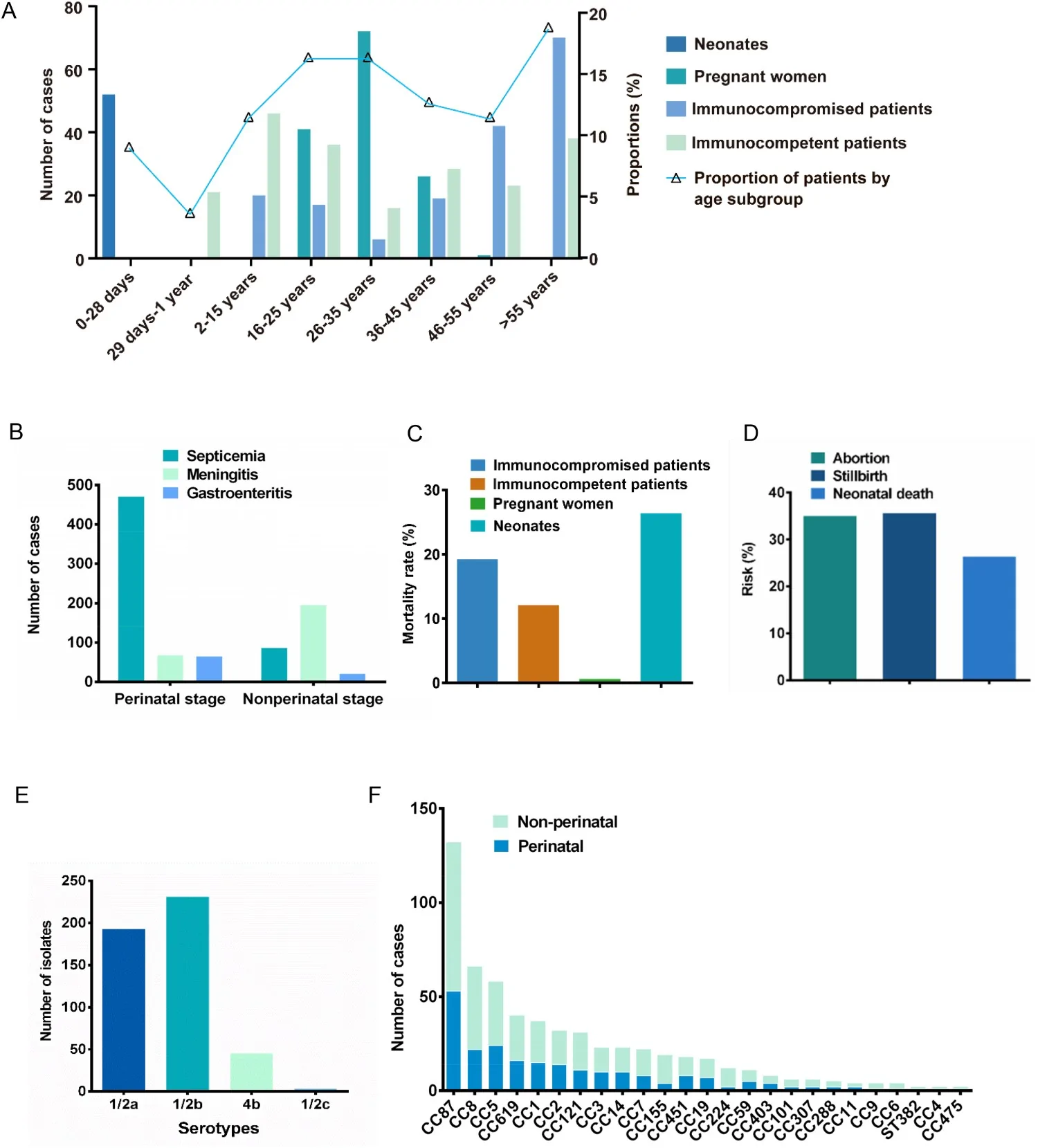
The epidemic features of human listeriosis infections. (A) Age distribution of population subgroups for listeriosis cases, n = 567; (B) the clinical symptoms of perinatal and non-perinatal infection; (C) clinical manifestations of perinatal and non-perinatal infection; (D) risk probabilities of clinical outcomes following maternal infection; (E) distribution of serotypes; (F) the distribution of the cloned polymorphic form.

#### Clinical manifestations and infection type

Among 322 non-perinatal Listeria infection cases with detailed clinical records, 60.6% were diagnosed with meningitis, 26.7% with sepsis, 8.4% with both meningitis and sepsis, and 6.7% with gastroenteritis (Figure 2B). In contrast, among 746 perinatal listeriosis cases, including 214 pregnant women and 532 neonates, sepsis was the most common manifestation (63.1%, 471/746), followed by bacteraemia (21.0%, 157/746), with central nervous system infections (9.0%, 67/746) and gastroenteritis (8.4%, 63/746) being less frequent (Figure 2B). Fever was the most common clinical symptom in perinatal infections, often accompanied by headache, altered mental status, nausea, vomiting, convulsions, and dyspnoea.

**Figure 3.**
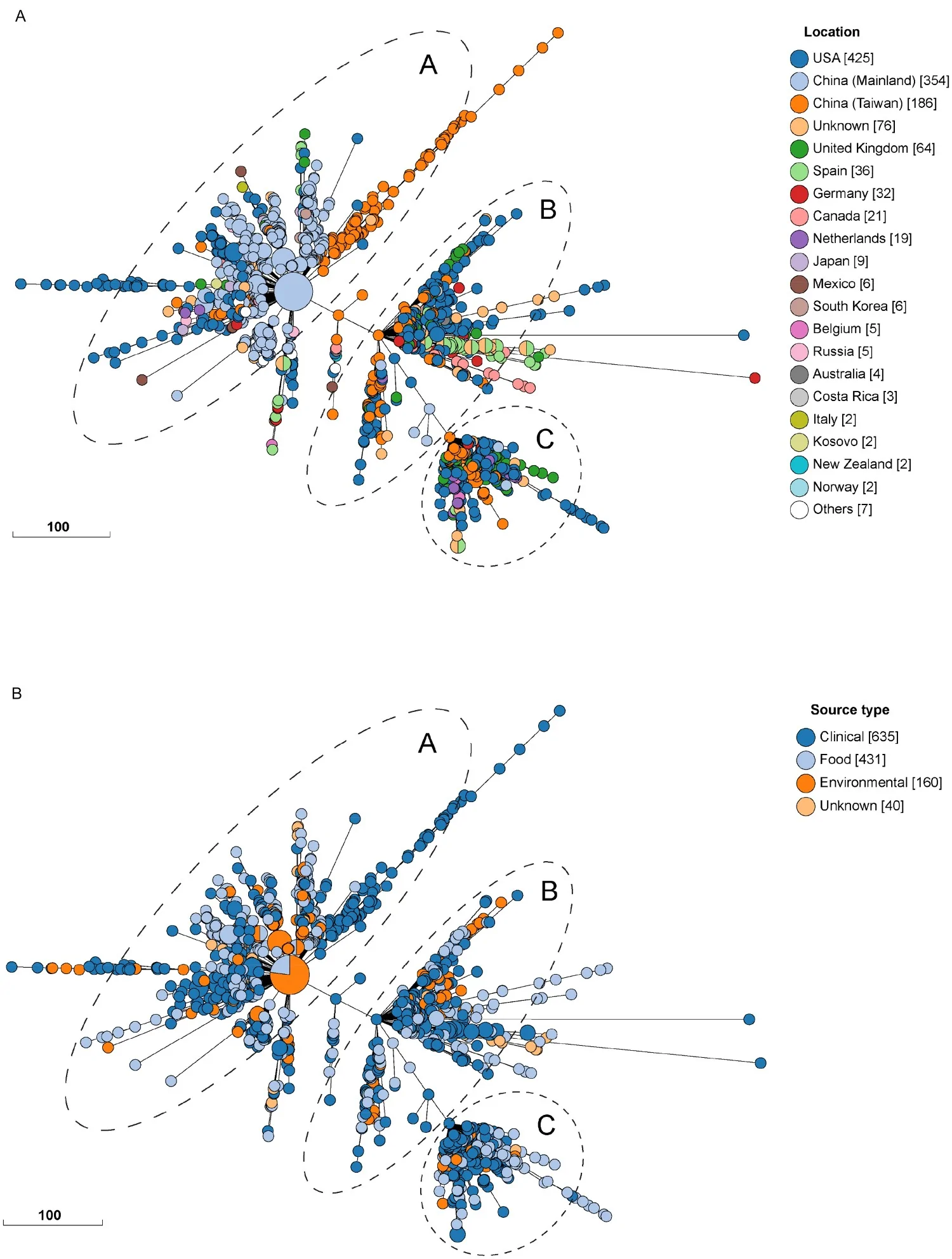
Phylogenetic analysis of global *L*. *monocytogenes* isolates and genomic diversity of CC87. (A) Minimum spanning tree based on cgMLST profiles of CC87 isolates from different countries; (B) the distribution of CC87 isolates from human, food, and environment.

#### Mortality and clinical outcomes

In non-perinatal cases, the overall case fatality rate was 12.1% (Figure 2C). Among 746 perinatal listeriosis cases with comprehensive clinical records, the overall case fatality rate was 33.0%. Among 309 neonates born to infected mothers, 134 developed listeriosis, with a mortality rate of 26.4% (Figure 2D). In the cohort of 214 pregnant women, fetal loss occurred in 25.7% (55/214), while only 5.1% (11/214) delivered healthy infants post-treatment (Figure 2D). Despite relatively low maternal mortality, the risk of fetal loss and neonatal infection remains substantial, highlighting pregnant women's heightened susceptibility to Listeria; timely screening and prevention are crucial for maternal and neonatal health.

### CC87 underlies perinatal and non-perinatal listeriosis

In this study, analysis of 473 clinically isolated strains with detailed records revealed that serotype 1/2b was the most prevalent (48.8%, 231/473), followed by serotype 1/2a (41.0%, 194/473), while serotypes 4b (9.5%) and 1/2c (0.6%) accounted for relatively lower proportions. These results indicate that serotypes 1/2b and 1/2a are the dominant epidemic serotypes causing human listeriosis in China (Figure 2E). Among the clonal complexes (CCs) with available data, CC87 was the predominant clone (23.0%), followed by CC8 (18.6%) and CC5 (17.3%) (Figure 2F). It is worth noting that CC87 is also the dominant clonal complex among food-derived isolates in China.

### The unique evolution and diversity of CC87

Given the predominance of CC87 among clinical isolates in China, we further investigated its evolutionary history and global population structure to better understand its genetic diversity and epidemiological success. A core genome multilocus sequence typing (cgMLST) analysis of CC87 genomes against a global collection of 1271 *L*. *monocytogenes* genomes revealed that CC87 isolates are primarily divided into three clades (A, B, and C) (Figure 3A). In contrast to isolates from other countries, most Chinese CC87 isolates formed a core part of the largest clade, Clade A. Notably, CC87 isolates from Taiwan, China, were distributed across all three clades. Conversely, most isolates from the United States were clustered into Clades B and C. This distinct phylogenetic separation by geographic origin underscores the substantial genetic diversity within the CC87. Furthermore, within the major Clade A, Chinese CC87 isolates from clinical, food, and environmental contexts were closely related (Figure 3B), suggesting a strong epidemiological link among these strains.

### The persistently circulating CC87 clone originated in the late 1960s

The global phylogeny of 646 representative CC87 isolates showed evidence of a temporal signal, as indicated by root-to-tip regression analysis demonstrating a correlation between collection dates and root-to-tip genetic distances (correlation coefficient = 0.408, R^2^ = 0.167). To further explore the evolutionary dynamics, a total of six population genetics models were compared through Bayes factors (BF) in the phylodynamic analyses. The combination of relaxed molecular clock model and a coalescent Bayesian Skyline population prior was favoured by yielding the highest BF among model combinations. The time of the MRCA was estimated using a Bayesian Markov chain Monte Carlo (MCMC) approach. The median date of MRCA for the CC87 strains was estimated to be around 1967.67 (95% HPD, 1956.00–1976.75) (Figure 4). The mean mutation rate across the CC87 population was inferred to be 1.99 × 10^−7^ substitutions per site per year or 0.59 SNPs per genome per year.

**Figure 4.**
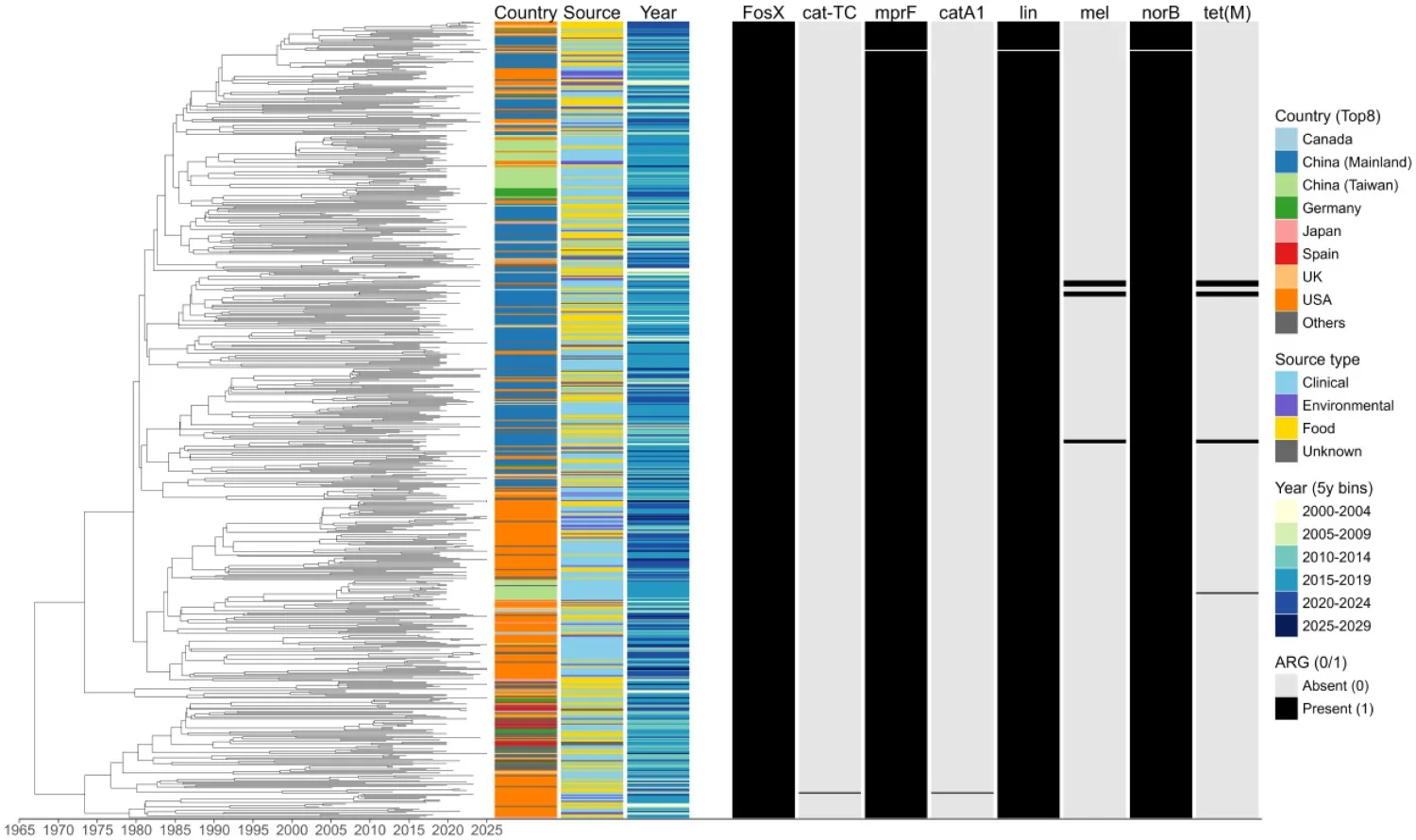
Global phylogeny and antimicrobial resistance gene distribution of *L*. *monocytogenes* CC87 isolates. A time-scaled phylogenetic tree of representative CC87 isolates reconstructed based on recombination-filtered polymorphic sites. The tree is annotated with metadata including country of origin, source type, and year of isolation (grouped in 5-year bins). The right panel shows the presence/absence (black = present, light grey = absent) of detected antimicrobial resistance genes. Countries are colour-coded for the top eight contributing regions, with remaining countries grouped as “Others.” Source types include clinical, environmental, food, and unknown. The scale bar represents time in years.

In addition, the distribution of antimicrobial resistance genes (ARGs) across the phylogeny revealed that several core resistance determinants, including *fosX*, *mprF*, *lin*, and *norB*, were highly conserved among global CC87 isolates. In contrast, a limited number of isolates sporadically acquired additional resistance genes, notably *mel* and *tet*(M), suggesting occasional horizontal gene transfer events during the global dissemination of this lineage. These observations are based on genomic data, which provide valuable insights into the potential for antimicrobial resistance; functional validation through phenotypic testing would further strengthen these findings.

### Nationwide circulation with a limited geographic structure of CC87

Phylogenetic analysis of CC87 isolates in China revealed a widespread distribution across multiple provinces, with isolates from diverse geographic regions extensively interspersed throughout the phylogeny (Figure 5A). Bayesian skyline plot analysis further revealed a gradual increase in the effective population size of CC87 since the late 1980s, followed by a sustained plateau after approximately 2000 (Figure 5B). This pattern suggests long-term nationwide expansion accompanied by stable persistence in recent years. Most major lineages comprised isolates from various provinces and sources (Figure 5A). Notably, Taiwan isolates showed partial phylogenetic aggregation, forming several localized sublineages, suggesting relatively stronger local circulation compared with the more widely mixed mainland population. Comparative analysis of virulence genes among these Chinese CC87 isolates revealed a highly conserved virulence profile (Figure 5C). Core virulence determinants, including LIPI-1 and LIPI-4, were present in nearly all isolates. Limited variability was observed in adherence and biofilm-related genes. Notably, the *comK* gene was frequently detected in isolates from Taiwan, suggesting possible regional enrichment. No clear clustering of overall virulence profiles was observed among clinical isolates.**Figure 5.**
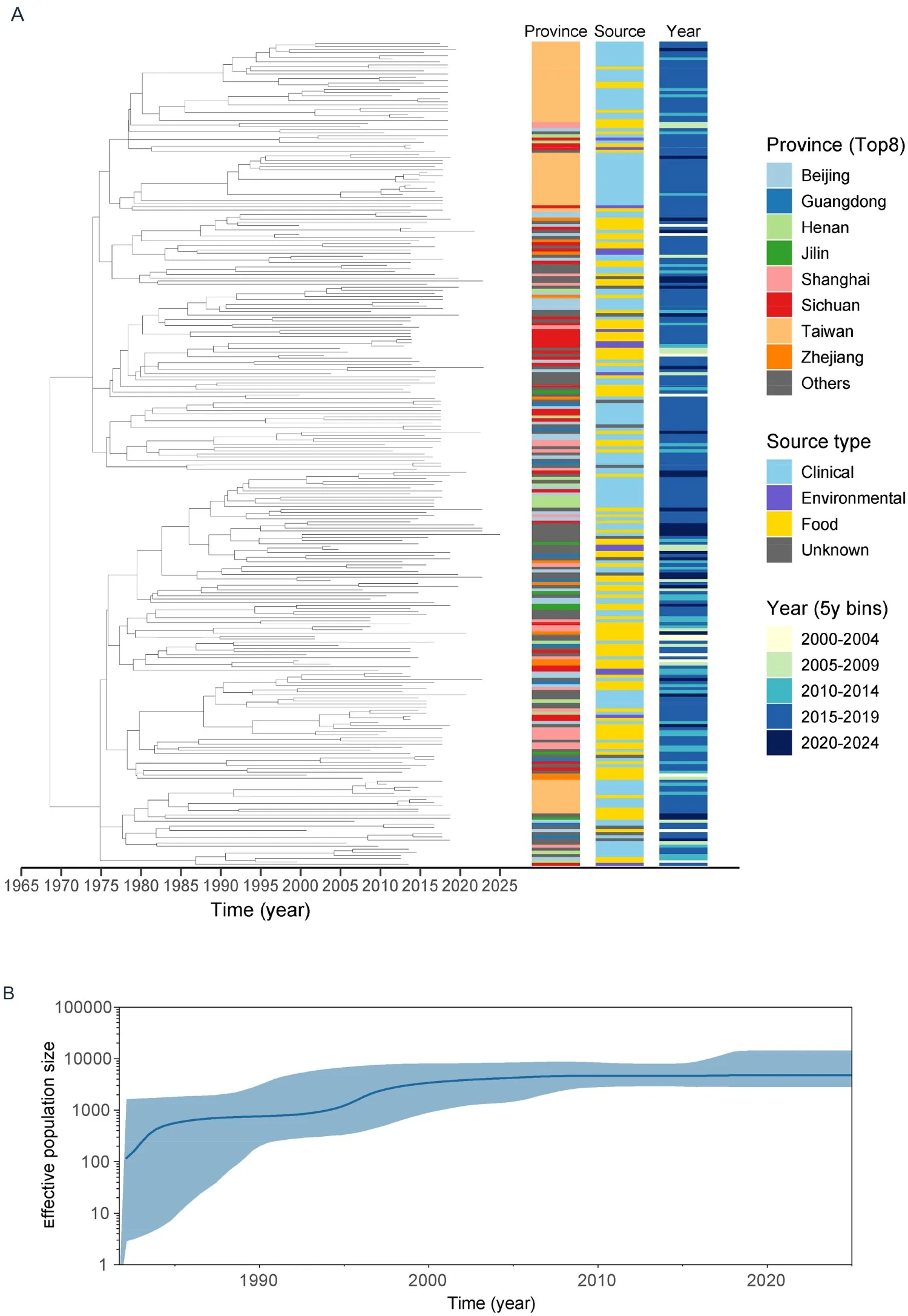
Phylogenetic structure and spatial genomic relatedness of *L*. *monocytogenes* CC87 isolates in China. (A) Time-scaled phylogeny of representative *L. monocytogenes* CC87 isolates from China. The phylogeny is annotated with metadata tracks indicating province of origin, source type, and year of isolation. (B) Bayesian skyline plot of the effective population size of CC87 over time. The solid blue line represents the median estimate, and the shaded area indicates the 95% highest posterior density (HPD) interval. (C) Virulence genes of representative *L. monocytogenes* CC87 isolates from China. The presence of genes is colour coded by virulence categories. In the phylogenetic tree, tip branch colours represent the source type, while node colours indicate the province of origin. (D) Pairwise core-genome SNP distance distributions within and between provinces among Chinese *L. monocytogenes* CC87 isolates. Violin plots show the distributions of pairwise core-genome SNP distances for CC87 isolates from China, comparing within-province and between-province pairs. Boxplots indicate medians and interquartile ranges. Dashed lines mark reference thresholds at 10, 20, and 50 SNPs. Only provinces with ≥10 isolates were included. E. Provincial pairwise core-genome SNP distance structure of clinical *L. monocytogenes* CC87 isolates in China. Heatmaps show the proportions of pairwise core-genome SNP distances between clinical CC87 isolates across Chinese provinces. Distances are grouped into three categories: <20 SNPs, 20–50 SNPs, and >50 SNPs. Each cell represents the proportion of isolate pairs between two provinces falling into the corresponding SNP distance bin. Only provinces with ≥3 clinical isolates were included.

**Figure 5.**
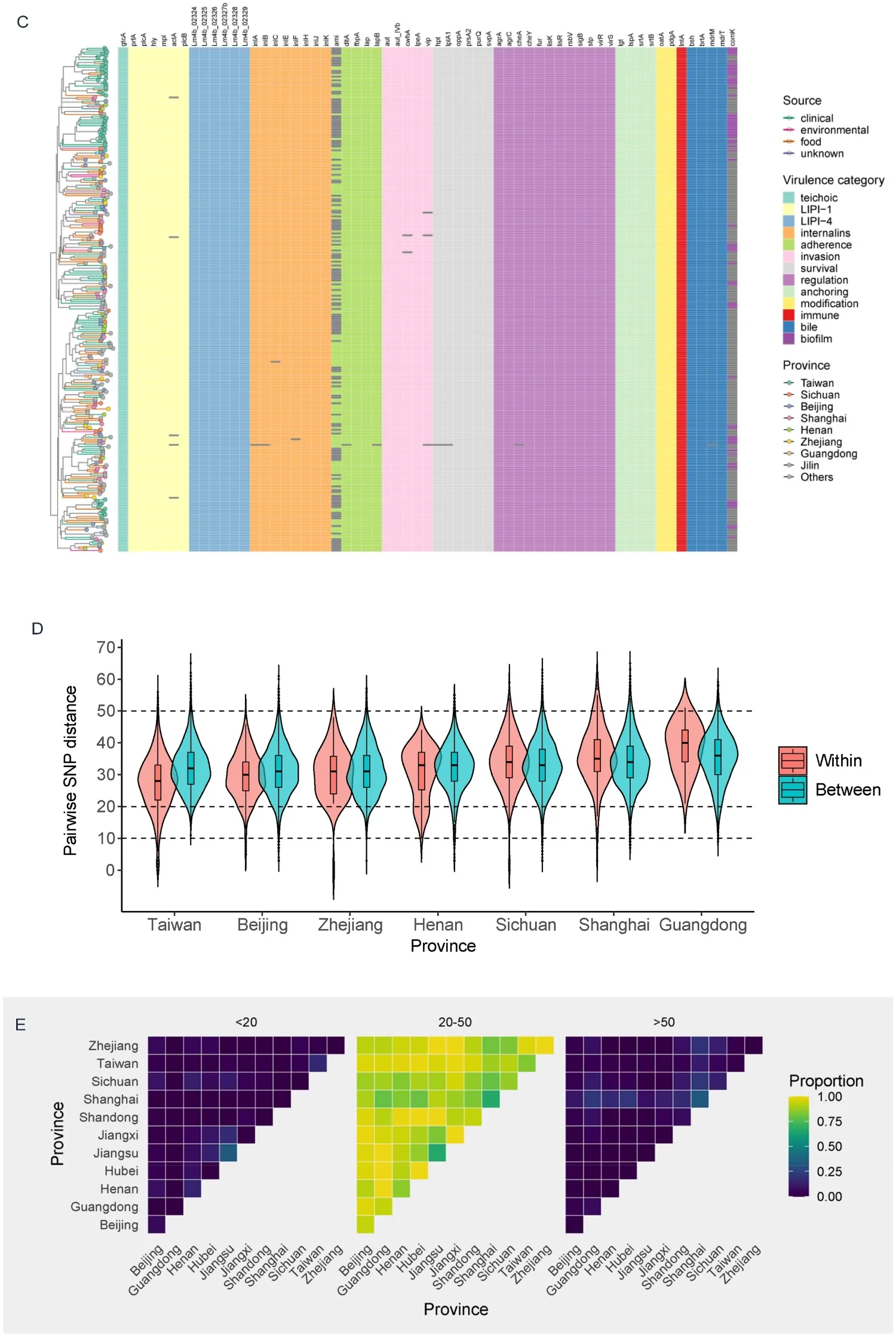
Continued

Pairwise core-genome SNP distance distributions showed extensive overlap between within- and between-province comparisons across regions (Figure 5D), indicating a weak geographic population structure. A small increase in between-province distances relative to within-province distances was detected in Taiwan and Beijing, while Guangdong showed the opposite pattern, with slightly higher within-province distances. Other provinces displayed nearly identical within- and between-province distributions. Overall, most pairwise distances clustered within the ∼20–50 SNP range. Based on the core-genome SNP distance matrix of clinical isolates, pairwise genomic distances between provinces were categorized into three bins (<20, 20–50, and >50 SNPs; Figure 5E). The SNP distances among clinical isolates were dominated by intermediate distances (20–50 SNPs) across provinces, with relatively few short-distance (<20 SNPs) or long-distance (>50 SNPs) pairs, indicating moderate genetic diversity and limited regional genetic structuring.

## Discussion

Listeriosis, a globally widespread foodborne illness caused by *L. monocytogenes*, continues to pose a persistent public health challenge despite advancements in food safety systems [20,27]. Understanding its epidemiological patterns in China is essential for developing sustained and effective control strategies. However, long-term spatiotemporal and molecular epidemiological data remain limited, particularly regarding the population dynamics of key clonal complexes such as the hypervirulent Clone Complex 87 (CC87), a predominant cause of invasive disease in China. Through a systematic review and comprehensive analysis of cases documented over six decades (–2020), we identified a three-phase trajectory: Era 1 (–2000), the Sentinel Period; Era 2 (2001–2010), the Growth Period; and Era 3 (2011–2020), the High-Incidence Plateau Period. We note that the apparent increase in reported cases may be partly influenced by changes in publication activity, reporting practices, and improvements in diagnostic capacity over time [20,27]. Nevertheless, by integrating a large number of published cases across multiple decades and regions, our analysis provides a robust overview of the long-term epidemiology of listeriosis in China, capturing trends that would be difficult to observe in smaller, localized studies. Notably, CC87 has persisted across China, forming a distinct evolutionary branch with nationwide circulation [28]. Overall, this six-decade record illustrates the transition of listeriosis in China from an underrecognized condition to a defined and persistent public health concern, while highlighting the importance of continued surveillance and research to inform control strategies.

The pathogenic potential of *L. monocytogenes* is shaped by the interplay between environmental fitness and the virulence gene repertoire of distinct clonal groups (CGs) [29,30]. Globally, Lineage I clonal complexes (CCs), including CC1, CC2, CC4, and CC6, are repeatedly linked to invasive listeriosis outbreaks [31,32], whereas in China, CC87 predominates among clinical isolates (29.6%), accounting for the highest proportion of perinatal infections, with CC8 more frequently associated with non-perinatal cases [24]. CC87 strains harbour both Listeria pathogenicity islands LIPI-1 and LIPI-4, enhancing intracellular survival and translocation across placental and blood–brain barriers [32]. Genomic and phylogenetic analyses indicate that the CC87 population in China exhibits a predominantly weak phylogeographic structure. The absence of pronounced geographical clustering and the substantial overlap between intra- and inter-provincial SNP distributions suggest limited geographic differentiation at the national scale. With a median pairwise distance largely within 20–50 SNPs, the population displays moderate genetic diversity, indicative of sustained evolutionary diversification rather than recent clonal expansion. Such spatial homogenization likely reflects ongoing cross-regional circulation across provinces, potentially facilitated by the movement of contaminated food products, livestock trade, or even human mobility. Temporal reconstruction places the most recent common ancestor (MRCA) of the Chinese CC87 population around 1969, indicating that this lineage has persisted in China for several decades. The Bayesian skyline plot further revealed a gradual expansion in effective population size beginning in the late 1980s, followed by stabilization at a relatively high level after 2000. Together, these findings point to a long-established, widely disseminated lineage of CC87 maintained through continuous circulation at the national scale. The core virulence determinants, including LIPI-1 and LIPI-4, are highly conserved across provinces, with minor variations such as *comK* being more frequent in Taiwan isolates. It should be noted that all isolates used for phylogenomic analyses were collected after 2000, which may limit the clock-like structure of the dataset. Accordingly, root-to-tip regression showed a modest temporal signal (R² = 0.18), suggesting that the estimated tMRCA may be biased towards more recent dates with associated uncertainty. Nevertheless, this measurable temporal signal still supports molecular clock-based inferences of evolutionary rates and relative divergence patterns.

The global dissemination and diversification of CC87 suggest that *L. monocytogenes* is continuously adapting to new environments, likely driven by selective pressures such as shifting local epidemiological factors [33,34]. Phylogenomic analyses further reveal that Chinese isolates form a distinct, geographically confined branch, indicating regional adaptation and endemic persistence [28]. In environments with appropriate antibiotic pressures, *L. monocytogenes* can acquire resistant phenotypes through sporadic mutations and gene acquisitions. The findings of this study reveal genetic determinants of antimicrobial resistance, including intrinsic resistance genes (*fosX*, *lin*, *norB*, *mprF*), with some isolates also carrying *tetM* and *mel*. Previous studies support the clinical relevance of such acquired resistance: Li et al. reported that several CC87 clones, along with Lineage II clones such as CC8/ST8 and CC155/ST705, have acquired multidrug resistance determinants [35]; Wang et al. found that CC87 strains showed reduced susceptibility to ampicillin (100%) and gentamicin (66.7%), underscoring significant therapeutic challenges and public health risks [36]. Minor regional differences in virulence genes highlight the value of continued genomic monitoring. These findings position CC87 as a highly adaptable strain with nationwide circulation and increasing genetic and phenotypic diversity, including evolving antimicrobial resistance over time. This research highlights the urgent need for continuous surveillance and targeted interventions to control its spread.

The development of China’s public health surveillance system has played a crucial role in shaping the understanding and management of listeriosis over time (Figure 6). During the Sentinel Period (–1999), limited infrastructure and laboratory capacity led to significant underreporting, with limited clinical cases documented between 1961 and 2000, and laboratory confirmation was scarce even during outbreaks. With the establishment of the Foodborne Disease Surveillance Network in 2001, and the TraNet platform in 2013, integrating data from 30 provinces and markedly enhancing national coverage and outbreak response capacity [37,38]. Regulatory measures were further strengthened through the 2014 National Food Safety Standard, which set a “non-detectable” limit for Listeria in ready-to-eat foods, and the 2015 Food Safety Law. A major milestone was reached in 2019 with the establishment of the national whole-genome sequencing (WGS) network, enabling precise linkage of sporadic cases to contamination sources. This capability was demonstrated by Niu et al., who traced a clinical isolate to a food-processing facility with only 1–2 cgMLST allele differences [39], while studies by Li et al. further highlighted the utility of WGS in resolving regional outbreaks and transmission chains [16,38]. Overall, the increase in case detection during Eras 2 and 3 reflects the combined effect of a modernized surveillance system and the adoption of advanced molecular typing, which together have revealed the previously underestimated burden of listeriosis in China [39].

**Figure 6.**
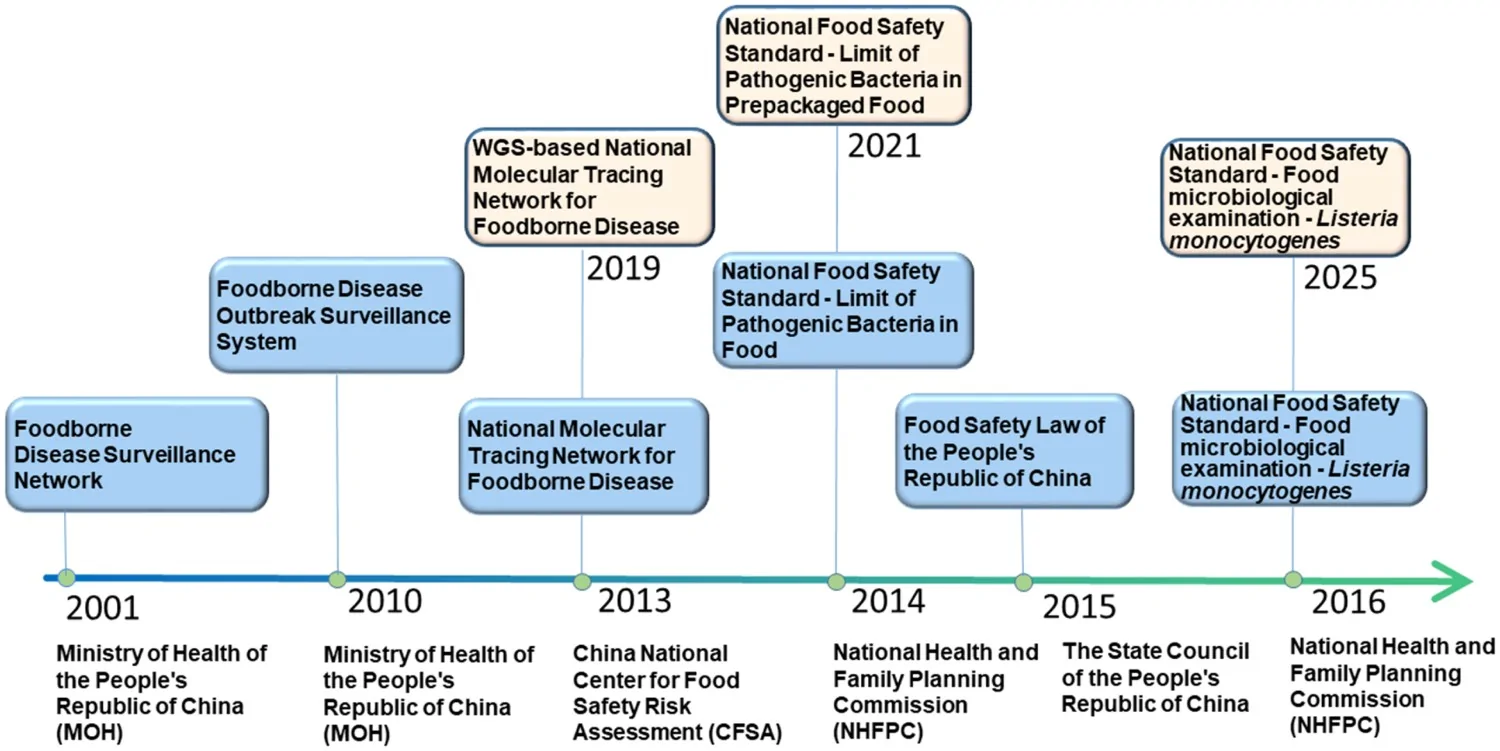
China's Food Safety Supervision and Administration System. Foodborne Disease Surveillance Network and the Foodborne Disease Outbreak Surveillance System were established in 2001 and 2010, respectively, by the Ministry of Health of the People’s Republic of China. The TraNet platform measures were established by the China National Center for Food Safety Risk Assessment in 2013. The National Food Safety Standard was promulgated by National Health and Family Planning Commission in 2014, which set a “non-detectable” limit for Listeria in ready-to-eat foods. The Food Safety Law was promulgated by the State Council of the People’s Republic of China in 2015, with a revised edition issued in 2021. The National Food Safety Standard for Food Microbiological Examination of *Listeria monocytogenes* was promulgated by the National Health and Family Planning Commission in 2016, with a revised edition issued in 2025. A major milestone was reached in 2019 with the establishment of the national whole-genome sequencing (WGS) network.

China's modernization, driven by shifts in dietary habits, urban lifestyles, and the growing demand for convenience, has significantly contributed to the rise of listeriosis [40,41]. Traditionally, *L. monocytogenes* contamination was eliminated through home cooking, but the expansion of the ready-to-eat (RTE) food sector, coupled with centralized food production in central kitchens, has exposed new vulnerabilities. RTE products, consumed without reheating, are directly at risk of contamination from raw materials to retail, bypassing the traditional cooking barrier and exposing a weakness in China’s food safety system [42,43]. Cold dishes and RTE meat products, particularly those containing mixed meats and offal, are major carriers, accounting for 44.5% of perinatal and 58.4% of non-perinatal cases [44]. Rising meat consumption, especially poultry, often a source of hypervirulent CC87 strains [45,46], amplifies the risk. Geospatially, economically developed provinces like Beijing, Sichuan, and Jiangsu report the highest burdens of listeriosis, reflecting both improved diagnostic capacity and socio-behavioural factors, such as reliance on convenience foods and regional culinary traditions. The incidence also peaks during warmer months, linked to increased consumption of cold dishes and RTE products, alongside shifts in China’s food ecosystem, including expanded cold chain use and year-round availability. Additionally, demographic transitions – such as an aging population and greater survival of immunocompromised individuals – expand the susceptible host pool [47,48]. These combined social, dietary, and demographic changes are key factors shaping the current epidemiology of listeriosis in China.

Our study relies on published reports and publicly available genomic sequences, which may introduce biases related to reporting practices, hospital coverage, and geographic representation. These data reflect the epidemiology of listeriosis in China prior to the establishment of the National Listeria Reference Center in 2013, which provides a more comprehensive and systematic surveillance framework. Nevertheless, integrating long-term, multi-region data allows us to capture temporal and geographic trends over several decades, offering valuable insights that would be difficult to obtain from shorter-term or single-centre studies.

## Conclusions

In summary, this temporal analysis over the past 60 years highlights a rapidly evolving listeriosis landscape in China, marked by increasing incidence associated with dietary modernization and enhanced public health surveillance. A key finding is the persistent dominance of the hypervirulent CC87, forming a distinct evolutionary branch in China and linked to severe clinical outcomes. Genomic analysis revealed the nationwide circulation of the CC87, with limited geographic clustering and ongoing diversification. While these findings support a “One Health” approach, the study mainly reflects regions with stronger healthcare and diagnostic capacity, providing a valuable baseline. Future work should prioritize prospective surveillance and molecular source-tracking to identify environmental reservoirs of CC87 and inform targeted control measures.

## Supplementary Material

Table S2.xlsx

Table S1.xlsx
