# Correction

**DOI:** 10.1080/22221751.2026.2740352

**Published:** 2026-09-29

**Article title:** Persisting predominant clinical CC87 Listeria monocytogenes in China: evolutionary dynamics and diversification

**Authors:** Huanga C., Qin X., Xu G., Xia R., Han H., Wang Z., Deng X., Jiao X., Li S., and Yin Y.

**Journal:** *Emerging Microbes & Infections*

**Bibliometrics:** Volume 15, Issue 1

**DOI:** https://doi.org/10.1080/22221751.2026.2688488

When this article was first published online, **Figure 2A contained an error**. During figure preparation, the data for pregnant women were inadvertently shifted by one row, resulting in incorrect alignment with the corresponding age groups (16–25, 26–35, and 36–45 years).

Importantly, this was a **figure preparation error** and does not affect the underlying data, statistical analysis, results, interpretations, or conclusions of the article.

The error has now been corrected. The corrected **Figure 2** is displayed below.

**Figure 2.**
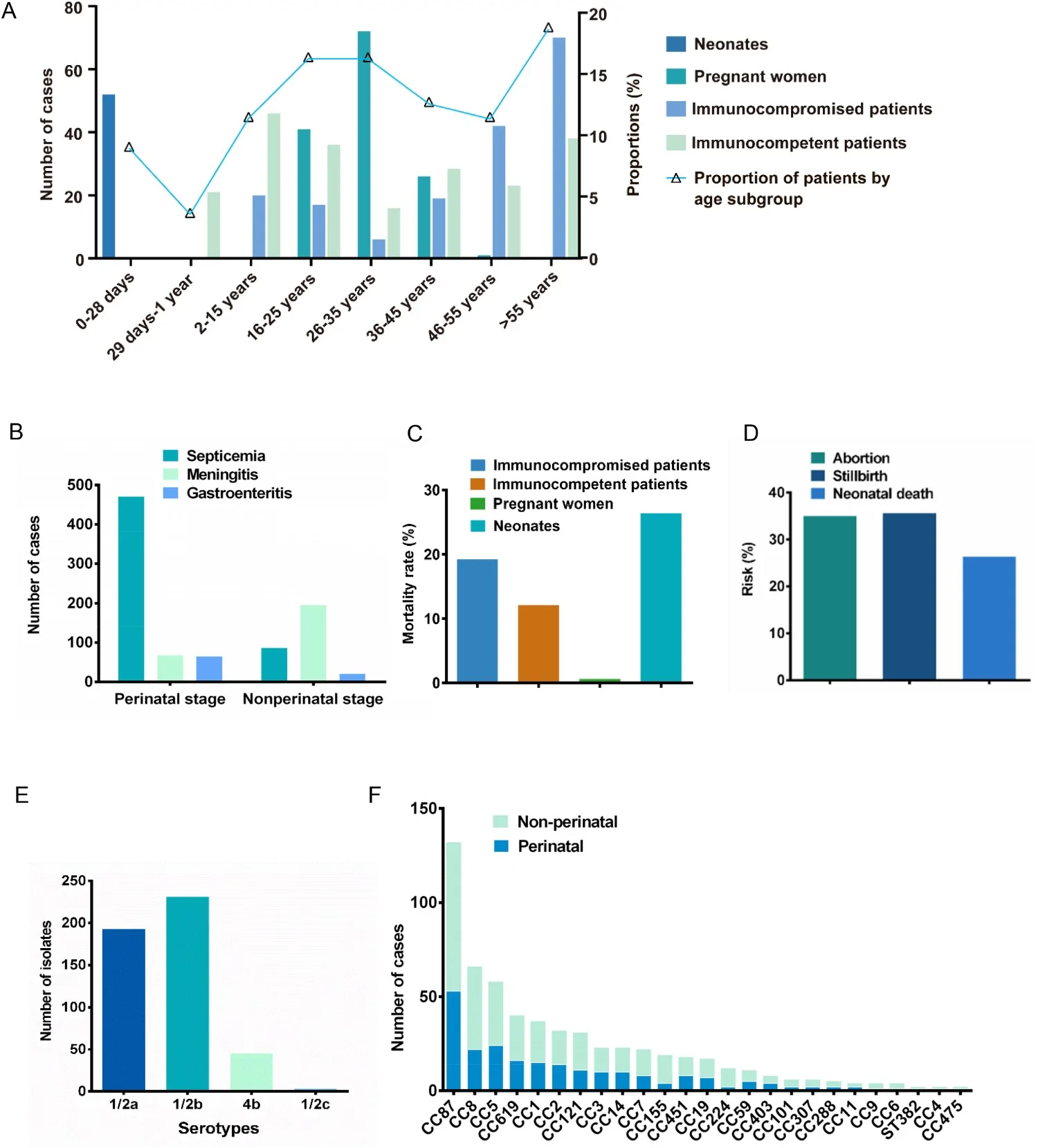
The epidemic features of human listeriosis infections. (A) Age distribution of population subgroups for listeriosis cases, n = 567; (B) the clinical symptoms of perinatal and non-perinatal infection; (C) clinical manifestations of perinatal and non-perinatal infection; (D) risk probabilities of clinical outcomes following maternal infection; (E) distribution of serotypes; (F) the distribution of the cloned polymorphic form.

